# Can I Work with and Help Others in This Field? How Communal Goals Influence Interest and Participation in STEM Fields

**DOI:** 10.3389/fpsyg.2017.00901

**Published:** 2017-05-31

**Authors:** Kathryn L. Boucher, Melissa A. Fuesting, Amanda B. Diekman, Mary C. Murphy

**Affiliations:** ^1^School of Psychological Sciences, University of IndianapolisIndianapolis, IN, United States; ^2^Department of Psychology, Miami UniversityOxford, OH, United States; ^3^Department of Psychological and Brain Sciences, Indiana UniversityBloomington, IN, United States

**Keywords:** communal goals, communal goal incongruity, STEM, gender, underrepresentation

## Abstract

Although science, technology, engineering, and mathematics (STEM) disciplines as a whole have made advances in gender parity and greater inclusion for women, these increases have been smaller or nonexistent in computing and engineering compared to other fields. In this focused review, we discuss how stereotypic perceptions of computing and engineering influence who enters, stays, and excels in these fields. We focus on *communal goal incongruity*–the idea that some STEM disciplines like engineering and computing are perceived as less aligned with people's communal goals of collaboration and helping others. In Part 1, we review the empirical literature that demonstrates how perceptions that these disciplines are incongruent with communal goals can especially deter women and girls, who highly endorse communal goals. In Part 2, we extend this perspective by reviewing accumulating evidence that perceived communal goal incongruity can deter *any* individual who values communal goals. Communal opportunities within computing and engineering have the potential to benefit first generation college students, underrepresented minority students, and communally-oriented men (as well as communally-oriented women). We describe the implications of this body of literature: describing how opting out of STEM in order to pursue fields perceived to encourage the pursuit of communal goals leave the stereotypic (mis)perceptions of computing and engineering unchanged and exacerbate female underrepresentation. In Part 3, we close with recommendations for how communal opportunities in computing and engineering can be highlighted to increase interest and motivation. By better integrating and publically acknowledging communal opportunities, the stereotypic perceptions of these fields could gradually change, making computing and engineering more inclusive and welcoming to all.

## Introduction

Sofia Tomov is a 12 year old computer programmer who developed an algorithm that helps doctors and their patients avoid adverse reactions to medication (Leins, 2016). She is just one of many young scientists who enter prestigious science competitions (e.g., Discovery Education 3M Young Scientist Challenge, Science Olympiad) each year with projects that showcase how science, technology, engineering, and mathematics (STEM) can be used to improve and save lives. Yet, when the lay public is asked to consider a typical engineer or a computer scientist or imagine engineering work, we are unlikely to think of exemplars like her.

Indeed, Sofia Tomov does not fit people's descriptions of computer scientists and engineers that include nerdy, socially awkward men who love science fiction and video games (e.g., Schott and Selwyn, 2000; Cheryan et al., 2009, 2013). Moreover, the applicability and relevance of contributions to people's lives are seldom emphasized in presentations and publications. In fact, STEM fields are rarely viewed as providing opportunities to improve lives or work with others, and, thus, the altruistic purposes that underlie so much of STEM work remain hidden (e.g., Diekman and Steinberg, 2013; Diekman et al., 2017). The hours of coding by computer scientists, the laboratory and teamwork required to develop a breakthrough method, and multiple fixes applied by engineers are not often linked to people's goals and motivations in an explicit way that makes those goals and motivations widely known. The result of these representations is that knowledge about what STEM is good for is underdeveloped. These representations lack the collaborative, real-world problem-solving that actually characterizes much of STEM work.

In the United States, shifting people's representations of STEM work to include scientists who emphasize collaboration in addition to innovation may be a key strategy to addressing a growing national concern. STEM fields are growing in numbers and are critical to the U.S. economy (e.g., U.S. Department of Education, 2006; National Academy of Sciences, National Academy of Engineering, and Institute of Medicine, Rising above the Gathering Storm Committee, 2010; Lennon, 2014). The need for more STEM professionals, particularly engineers and computer scientists, is acute. Developing strategies to recruit groups that are currently underrepresented in STEM, including female students, as well as Black, Hispanic, and Native American students, will play an important role not only in diversifying the STEM workforce, but in growing it to the size necessary to compete in the global economy.

Stereotypic perceptions of STEM fields are one of several important factors that explain patterns of group underrepresentation in STEM fields (e.g., American Association of University Women, 2010, 2015; Cheryan et al., 2017). Indeed, research shows that by showcasing how STEM fields can fulfill people's communal goals of collaboration and helping others, women's STEM interest and participation can increase, and these education efforts may lead to revisions in the stereotypes of who excels and belongs in STEM fields (e.g., Diekman et al., 2011). An underexplored implication of this research is that we may be losing many people–both women *and men*–who wish to be engaged in STEM but who *also* wish to be engaged in careers that make a difference to people's lives.

These communally-oriented individuals are likely to be essential in changing STEM stereotypes and STEM environments to be more welcoming and inclusive to all. When more communally-oriented men leave STEM fields (or decide not to pursue these fields because they do not see opportunities to fulfill their communal goals through STEM), the stereotypes of these fields as predominantly masculine and less communal are maintained and will become harder to change even if greater numbers of women are recruited and retained in STEM. In order to change perceptions of STEM and increase the STEM workforce, we will need more women *and* men whose work and stated purpose relate to communal motives. In this review, we discuss the research that supports this possibility.

## Focus of the current review

Our review proceeds in three parts. In Part 1, we first provide an overview of the communal goal perspective, with particular attention to *communal goal incongruity*—the idea that some STEM disciplines like engineering and computing are perceived as less likely to offer opportunities to meet communal goals. That is, when it comes to the “doing” of STEM work, these fields are seen as less collaborative and less centered on helping others. We focus on communal goals because communion and its counterpart agency are posited as the core human motivations necessary for optimal functioning (Bakan, 1966). Communal motivation involves a drive to connect, care, and share with others, whereas agentic motivation involves concerns of the self in terms of one's status, achievement, and independence. Communion and agency are key dimensions for judgments of the self and others (e.g., Judd et al., 2005; Abele and Wojciszke, 2007; Fiske et al., 2007).

Moreover, these goals serve as a lens for how people view and choose different environments like STEM fields (e.g., Diekman and Eagly, 2008; Diekman et al., 2017). When making the decision to enter, stay, or leave a setting, people draw upon beliefs about whether a role will allow them to pursue goals (i.e., *goal affordances*) to determine which of their valued goals can be accomplished (or might be impeded) within the setting. When there is a match between the environment and our goals, we experience *goal congruity*. Goal congruity prompts a state of “motivational readiness” and a pleasant sense of fit (e.g., Kruglanski et al., 2014), whereas goal incongruity can inspire questions about how to achieve congruity, and this search often leads us to change or leave the environment. Thus, communal goal incongruity has been posited as an explanation for differences in STEM interest and success, and this evidence will be reviewed in this part.

When available, we include evidence of communal goal incongruity for individuals in computer science and engineering, as women remain especially underrepresented in these STEM disciplines (e.g., National Science Foundation, 2015; National Science Board, 2016). Next, we extend this perspective to understand how communal goal endorsement and beliefs about communal opportunities in STEM might deter other groups from pursuing STEM (e.g., underrepresented minority students, communally-oriented men, and first generation college students; Part 2). Finally, our review closes with recommendations for offering and highlighting communal opportunities in computing and engineering (Part 3).

This review builds upon existing and emerging syntheses of the empirical literature. Two recent systematic reviews of gender differences in STEM have focused on the role of stereotypic perceptions in shaping gendered STEM participation. Cheryan et al. (2017) provide a model of the contributing factors to gender participation gaps in varying STEM fields, noting stereotypes of the fields as one key contributor. Diekman et al. (2017) review the existing evidence for this particular factor in relation to how STEM fields are stereotyped as less communal than other fields. Building from these syntheses, we conducted a more focused review to further elaborate the communal goal incongruity perspective. Specifically, we extend this perspective to focus beyond gender differences.

Past goal incongruity research has highlighted relative gender differences in the endorsement of communal goals and the impact of this endorsement on women's participation in STEM (e.g., Diekman et al., 2011). However, as with other psychological variables, a focus on gender difference can obscure the gender similarities that exist as well (Hyde, 2005). In this review, we thus examine how similar communal goal processes can explain the STEM decisions of a wide range of people from many different social groups. For example, accumulating evidence suggests that communal goals are highly valued for other group memberships besides women, yet there has not been an attempt to integrate these findings with those examining communal goal incongruity for women in STEM. Thus, our focused review has these two aims. We review new and accumulating evidence that suggests that perceived communal goal incongruity can deter *any* individual who values communal goals. This extension, thus, leads to the final novel contribution of our review, which is to focus on implications of communal goal incongruity for stereotype change and for interventions targeted to multiple groups. Making known the communal opportunities within STEM has the potential to benefit all communally-oriented people, including first generation college students, underrepresented racial and ethnic minority students, and communally-oriented men—as well as communally-oriented women.

To conduct this focused review, we obtained research through computerized literature searches via psychology (PsycINFO) and education (ERIC) databases. We restricted our search to published empirical papers that included the measurement of goals or motivation for both an underrepresented group in STEM (e.g., women, racial minorities) and a represented group in STEM (e.g., men, Whites). To examine similarities and differences for men and women in terms of communal goal endorsement, the following search terms were used: “gender differences” or “men AND women” with combinations of “goals,” “motivation,” “commun^*^,” “STEM,” “science,” and “math^*^.” Search results were similar when using “STEM,” “science,” or “math^*^.” To examine similarities and differences for different racial and ethnic groups in terms of communal goal incongruity, the following search terms were used: “racial differences,” “ethnic differences,” “race,” or “ethnicity” with combinations of “goals,” “motivation,” “commun^*^,” “STEM,” “science,” and “math^*^.” Again, search results were similar when using “STEM,” “science,” or “math^*^.” Lastly, since papers examining first generation college student status came up in multiple searches, we used similar keyword searches for this group.

With the resulting citations from our literature searches, we first examined the titles for relevance and redundancy. If papers explored group differences in goals or motivations in academic contexts, we then reviewed the abstracts and main text to see if the paper's method and results fit the aim of our focused review: reviewing empirical evidence of differences in goals or motivation for underrepresented and better represented groups in STEM fields. Published papers that examined possible group differences in goals or motivation, particularly those relating to communion, are included in this focused review (see Table 1 for included papers with a focus on gender and Table 2 for included papers with a focus on race/ethnicity and first generation status).

**Table 1:** Included studies in the focused review with a focus on gender.

**Citations**	**Study characteristics**	**Relevant findings**
Morgan et al., 2001	College students	Women were more likely to report that they planned to pursue a career because it was people oriented. Physical and mathematical sciences were seen as involving others to a lesser extent that careers in medicine or education.
	Cross-sectional	
	Work goals for careers including STEM	
Evans and Diekman, 2009	College students	Male stereotypical fields were seen as deficient in caregiving affordances but high in status affordances, while female stereotypical fields were seen as the opposite. Women were more likely to endorse caregiving goals, and men were more likely to endorse status goals. Gender differences in goal endorsement mediated gender differences in interest in these careers.
	Cross-sectional	
	Goal affordances and gender representation in fields including STEM	
Diekman et al., 2010	College students	STEM was seen as uniquely impeding communal goal pursuit, and communal goal endorsement negatively predicted STEM interest and mediated gender differences in STEM interest.
	Cross-sectional	
	Communal goal endorsement in STEM	
Diekman et al., 2011	College students	STEM is explicitly and implicitly stereotyped as lacking in communion. Activating communal goals resulted in lower interest in STEM careers, but not lower interest in non-STEM careers. Highlighting how science affords communal goals particularly increased women's positivity toward science careers.
	Cross-sectional and experimental	
	Communal stereotypes and goal activation in STEM	
Klotz et al., 2014	College-aged women and men Cross-sectional Communal goals in engineering	Students who weren't in engineering in comparison to engineering students were significantly less likely to believe that sustainability was associated with engineering. Believing that engineering could help save lives or improve quality of life significantly predicted intention to pursue an engineering career above and beyond gender and grades in math.
Brown et al., 2015a	College students	Increasing science communal affordances increased research positivity and science career motivation; highlighting agentic affordances did not increase these outcomes.
	Cross-sectional and experimental	
	Goal affordances in biomedical science	
Brown et al., 2015b	College students	Controlling for STEM major status, communal goal affordances predicted STEM career interest, research task positivity, and career motivation. Beliefs that STEM afforded communal goals predicted science motivation 10–12 weeks later.
	Cross-sectional and longitudinal	
	Communal affordances in STEM	
Clark et al., 2016	College students	Increasing science communal affordances increased science career positivity for men and women with male and female scientists.
	Cross-sectional and experimental	
	Highlighting communal affordances in science	
Stout et al., 2016	College students	Women who perceived physicial sciences as affording more communal goals at Time 1 took similar amounts of pSTEM courses as men 3 years later. Women who viewed pSTEM as deficient in communal affordances took fewer pSTEM courses than men 3 years later.
	Longitudinal	
	Gender roles and STEM stereotypes	
Fuesting and Diekman, 2017	College students	Participants viewed it as more challenging to locate communal STEM role models than communal role models in other fields. Women and men preferred to work with potential STEM advisors that exhibited communal characteristics.
	Cross-sectional and experimental	
	Communal role models in STEM	

**Table 2:** Included studies in the focused review with a focus on race/ethnicity and first generation status.

**Citations**	**Study characteristics**	**Relevant findings**
Fryberg and Markus, 2007	American Indian, Asian American, and European American college students at mainstream and tribal universities	American Indian college students mentioned helping one's communities more and had more negative associations with education. American Indian college students emphasized family and community concerns more than academic concerns. American Indian and Asian American college students endorsed both independent and interdependent self-representations, while European Americans only endorse independent ones.
	Cross-sectional	
	Endorsement of interdependent selves and views of education	
Stephens et al., 2012	University administrators and first generation and continuing generation college students	Universities have norms that primarily emphasize independence. There is a mismatch between universities' emphasis on independence with first generation students' goals of interdependence, and this mismatch predicts lower grades. Goal mismatch undermined first generation college students' performance relative to goal matching for interdependence.
	Cross-sectional, longitudinal, and experimental	
	Assessing university cultural norms of interdependence and independence and their impact on students	
Fryberg et al., 2013	Native American and European American high school students	Native American students showed more interdependent self-representations and less trust for teachers, and interdependent self-representations and teacher trust were positively related to their academic performance.
	Cross-sectional	
	Endorsement of independent and interdependent selves and impact on performance and trust	
Smith et al., 2014	Native American and White American college freshmen	Native American STEM freshmen (men and women) more highly endorsed communal work goals. Communal goal endorsement predicted belonging uncertainty, lower motivation, and perceived poor performance in a later semester.
	STEM and non-STEM majors	
	Cross-sectional and longitudinal	
	Communal goal incongruence in STEM	
Allen et al., 2015	Generation status (first and continuing) at a minority-serving institution	First generation college students who believe science can afford communal goals are more interested in science. First generation college students want to stay closer to home for graduate education, and higher communal goal orientation predicted this tendency to want to stay closer to home. Race/ethnicity did not explain the patterns for either study for first generation college students.
	College students and research assistants in science	
	Cross-sectional and longitudinal	
	Work, communion, and agentic goals, and science interest	
Covarrubias and Fryberg, 2015	Latino and White college students of first and continuing generations	First generation college students have greater family achievement guilt, with Latino first generation students reporting the most. First generation college students who reflected on a time they helped their family reported less family achievement guilt.
	Cross-sectional and experimental	
	Highlighting communal behavior and its impact on family achievement guilt for attending college	
Thoman et al., 2015	Underrepresented racial minorities in STEM and those from well-represented groups	Underrepresented minority student RAs who saw altruistic values in their research felt more involved and interested in science over time.
	College students working in biomedical labs at universities and tribal colleges	
	Longitudinal	
	Communal values and science research interest	
Harackiewicz et al., 2016	Race/ethnicity (underrepresented minority and not) and generation status (first and continuing)	The utility value intervention was particularly successful in reducing the achievement gap for first generation, underrepresented racial minorities in the biology course.
	College students in biology course	
	Longitudinal	
	Utility value intervention in biology	

## Part 1. Overview of evidence for communal goal incongruity processes as a deterrent in STEM

Individuals can have multiple motivations for pursuing or not pursuing STEM fields, and these fields can be seen as affording multiple goals as well. Decisions can be based upon the perceived value and earning potential of and mobility within STEM occupations. Work-life balance may also be a goal that is considered in occupational choice (Ceci et al., 2009; Weisgram and Diekman, 2015). Although these goals are important, goals of communion and agency within the work itself may be critical for STEM occupational choices because STEM fields are often perceived as unbalanced in their ability to afford both communal and agentic goals. Goals of communion and agency, although sometimes viewed as opposing motivations, can co-exist; however, as we review below, STEM fields are perceived as affording greater opportunities for agency than communion.

Out of the various goals that people can pursue (e.g., work-life balance, financial security, mobility, status), communal goals are central to this review for several reasons. First, communal goals are beneficial for the individuals pursuing them and those who may benefit from their work. Pursuing communal goals helps fulfill needs for belonging (e.g., Baumeister and Leary, 1995; Fiske, 2004), connection (e.g., Deci and Ryan, 2000), and affiliation (e.g., Hill, 1987). Moreover, communal goals and activities are related to positive outcomes, such as greater social support, feeling more positive emotions, and less stress (e.g., Crocker and Canevello, 2008; Poulin et al., 2013; Poulin, 2014). These benefits extend to the workplace as collaboration with colleagues predicts scientific productivity (e.g., Landry et al., 1996; Lee and Bozeman, 2005) and having closer contact with people one helps at work relates to greater motivation and persistence in the workplace (e.g., Grant, 2007; Grant et al., 2007).

Secondly, stereotypes of communal goal affordances vary by occupational field. Regardless of whether it reflects the reality of these careers, people tend to perceive some careers as fulfilling more communal goals than other careers. STEM fields, and computing and engineering in particular, are viewed as fulfilling fewer communal goals than a wide range of other fields but are viewed as fulfilling similar agentic goals as other traditionally male-dominated fields (Diekman et al., 2011). Agentic motives are naturally important to achievement and success in STEM (e.g., Diekman et al., 2017) and may also help individuals persist through the education necessary to pursue STEM careers. However, it is unlikely that highlighting how STEM fulfills self-oriented goals will make these fields more appealing; most individuals are already well aware how STEM can provide opportunities to pursue self-oriented goals, and highlighting STEM agentic affordances will only confirm individuals' beliefs about STEM. Alternatively, highlighting how STEM affords communal goals disrupts prevalent stereotypes about STEM and should, thus, increase interest in these careers.

Finally, as detailed below, there is also variability in communal goal endorsement across individuals, and members of groups underrepresented in STEM fields (i.e., women, racial and ethnic minorities, and first generation college students) tend to value communal goals to a greater extent than members of groups that are better represented (e.g., Evans and Diekman, 2009; Stephens et al., 2012; Smith et al., 2014; Harackiewicz et al., 2016). Taken together, a focus on communal goals can result in a better understanding of patterns of underrepresentation and ways to recruit and retain more diverse groups of STEM majors and workers.

### Perceived goal affordances in STEM

In the United States, stereotypes portray STEM fields as agentic occupations in which goals of independence and status can be pursued; however, these stereotypes also portray STEM fields as providing fewer opportunities than non-STEM fields to pursue communal goals. For instance, male and female undergraduates report that the physical sciences and mathematics allow fewer opportunities to work with and help others than careers in education, the social sciences, or medicine (Morgan et al., 2001). A similar pattern emerges when considering how STEM fields like engineering and computer science specifically relate to non-STEM occupations that are viewed as stereotypically male or female. Specifically, young men and women, both in STEM and other majors, view STEM occupations (e.g., engineering, computer science, and environmental science) as fulfilling fewer communal goals than stereotypically male non-STEM occupations (e.g., business) and stereotypically female non-STEM occupations (e.g., nursing; Diekman et al., 2010, 2011).

Looking within the subdisciplines of STEM, there is consistent evidence across multiple studies that computer science and engineering are viewed as even less communally oriented than many other STEM fields, a view reported by men and women. Careers in engineering and computer science (along with those in physical science and mathematics) were more strongly associated with agentic goals like self-direction and self-promotion than with communal goals (Stout et al., 2016). Other science-intensive careers in the biological and social sciences (e.g., anthropology, psychology) that exhibit more gender parity were conversely perceived as fulfilling more communal than agentic goals (Stout et al., 2016).

Due to widespread stereotypes that computing and engineering allow individuals fewer opportunities to pursue communal goals, a communally-oriented person can feel a lack of fit. The mismatch between what these fields afford and what motivates a person can lower people's motivation to pursue these fields. For example, valuing communal goals negatively predicts interest in engineering, environmental science, and computing even for people who feel that they are good at STEM (Diekman et al., 2010). It follows that these STEM fields may be less likely to attract people who highly value communal goals, many of whom belong to groups currently underrepresented in STEM (e.g., women, racial and ethnic minorities; Diekman et al., 2017). Members of these groups may opt out of STEM in favor of more communally-oriented fields in which they excel and that do not involve negative group stereotypes (e.g., Eccles, 1994; Wang et al., 2013).

### Communal goal incongruity is a barrier for women in STEM

Past research on communal goal incongruity has largely focused on how perceived goal incongruity can deter members of groups who are currently underrepresented in STEM because communal goals are especially valued by these groups. Women, on average, tend to value communal goals more strongly than men (Evans and Diekman, 2009; Diekman et al., 2011). If STEM environments are perceived as not valuing and not capable of fulfilling communal goals, then communal goal incongruity may reduce women's motivation to pursue STEM (see Diekman and Steinberg, 2013; Diekman et al., 2017, for further review). Conversely, fields that are perceived to afford communal goals attract people who are communally-oriented, and the experience of fit due to goal congruity can result in greater interest and motivation in these fields. In this way, communally-oriented women may be pulled into non-STEM fields in addition to being pushed out of STEM fields.

Correlational evidence from Morgan et al. (2001) found that compared to men, women placed greater importance on interpersonal work goals, perceived STEM fields like the physical sciences and mathematics to be less interesting, and were less likely to report plans to work in these fields. Importantly, experimental evidence shows that communicating that STEM fields can fulfill communal goals boosts women's STEM interest. For instance, women who learn that a scientist spends the day working with others (as opposed to working alone) exhibited particularly strong increases in their attitudes toward science careers (Diekman et al., 2011, Experiment 3).

Extending these findings beyond the lab, communal goal (mis)match relates to students' actual course taking behavior (Stout et al., 2016). This research demonstrated that gender differences in STEM college course taking stemmed from individuals who believe that engineering and computing do not fulfill communal goals: women who believed that engineering and computing fulfilled few other-oriented goals took fewer computing and engineering courses than men with similar beliefs. However, among students who believed that engineering and computing fulfilled relatively more communal goals, there was no gender gap in course taking. Men and women who similarly believe that computing and engineering fulfill communal goals demonstrate similar motivation to pursue computing and engineering courses. Therefore, differences in perceived communal opportunities can play a part in who is attracted to and persists in STEM. Promoting communal opportunities in STEM can increase the number of communally-oriented people in STEM. Without the increased representation of these individuals, stereotypes of these fields as less communal are likely maintained.

## Part 2. Extensions of the communal goal incongruity perspective to other groups

### Communal goal incongruity is a barrier for other underrepresented groups in STEM

Although research on communal goal incongruity has mainly explored the impact of anticipated goal fit for women in STEM, other research has focused on communal goal processes for members of underrepresented racial/ethnic groups in STEM. Here, we integrate this work into the broader literature on communal goal incongruity in STEM contexts.

Black, Hispanic, and Native American students, both male and female, have less access to advanced STEM courses in high school (e.g., May and Chubin, 2003; Tyson et al., 2007; Perna et al., 2009) and are less likely to enter and receive degrees in STEM fields (e.g., National Science Foundation, 2015). Racial and ethnic minority students in STEM fields, especially in computing and engineering, continue to be underrepresented at all levels of these disciplines (e.g., American Association of University Women, 2010). This underrepresentation extends into the workplace as racial and ethnic minorities make up a small proportion of workers in STEM fields (e.g., U.S. Census Bureau, 2011).

Communal goal incongruity can influence underrepresented racial and ethnic minority students' overall success in college. Perceiving the culture of academia as one that emphasizes and rewards assertiveness and independence over connectedness and interdependence can lead to a perceived mismatch for racial and ethnic minority students; this mismatch can reduce belonging and impair performance in college (e.g., Fryberg and Markus, 2007; Fryberg et al., 2013). Moreover, research shows that this mismatch can lower well-being and impede the academic success of first generation college students—individuals who are the first member of their family to pursue college (e.g., Stephens et al., 2012; Covarrubias and Fryberg, 2015).

Seeing STEM fields in particular as less communal also negatively influences members of these groups who tend to highly value communal goals (e.g., Smith et al., 2014; Harackiewicz et al., 2016). Researchers have, thus, investigated whether communal goal incongruity reduces underrepresented minority students' STEM motivation. Native American STEM students exhibited less motivation to complete their majors and degrees if they valued communal goals (Smith et al., 2014). Moreover, beliefs that science fulfills altruistic goals predict increased science career motivation especially among students from underrepresented ethnic backgrounds (Thoman et al., 2015). Similarly for first generation college students, beliefs that science allows people to help others predict their science course interest, suggesting that perceptions that science does not fulfill communal goals may be particularly demotivating for first generation college students (Allen et al., 2015).

Communal goal congruity has emerged as a possible mechanism to enhance the full participation and success of students from diverse backgrounds in STEM fields. Engaging collaborative and altruistic motivations can yield benefits across different, underrepresented group memberships because these motives are highly valued and these opportunities are perceived as extremely scarce or nonexistent. Future research is needed to delineate these patterns within specific STEM disciplines like computer science and engineering and explore whether the content of communal goals and the interventions to highlight them will work for all underrepresented groups that value these goals.

### Communal goal incongruity can also push men out of STEM

Because members of underrepresented groups are more likely to be negatively affected by communal goal incongruity, most communal goal incongruity research focuses on the implications of noncommunal STEM stereotypes for recruiting and retaining a more diverse STEM student body and workforce. Developing strategies to increase and sustain motivation to pursue STEM careers among underrepresented groups in STEM is increasingly important and essential. However, *all* individuals who value communal goals—not just members of underrepresented groups in STEM—may be negatively influenced by communal goal incongruity.

Focusing on the relative group differences in communal goal endorsement between men and women can obscure that men also value communal goals. Although men exhibit lower averages than women, men still show relatively high levels of communal goal endorsement (e.g., Evans and Diekman, 2009; Diekman et al., 2011), demonstrating that men, too, value communal goals. If communally-oriented men perceive STEM environments as not valuing and not capable of fulfilling communal goals, they—like women—can experience the negative effects of communal goal incongruity (Diekman et al., 2017).

When communal goals are activated, both men and women exhibit reduced STEM interest in comparison to when communal goals are not activated (Diekman et al., 2011, Experiment 2). If people believe that science careers fulfill communal goals, both men and women who value communal goals exhibit more positive attitudes toward science careers (Diekman et al., 2011, Experiment 3). Individual differences in communal goal endorsement—and not group membership in and of itself—thus determines who will be most affected by communal goal incongruity (Diekman et al., 2011, 2017).

Relatedly, fostering congruity between one's personal goals and the environment can increase men and women's favorability toward science careers and their motivation to pursue them. Regardless of gender, beliefs that STEM fulfills communal goals predict undergraduates' increased motivation to pursue a science career (Brown et al., 2015b). In several experiments, both men and women exhibited increased science career motivation when they learned how scientific research fulfills other-oriented goals (Brown et al., 2015a). Moreover, learning that scientists integrate collaboration into their workdays—instead of learning that scientists spend their days working mostly alone—increases both men and women's attitudes toward pursuing a science career (Clark et al., 2016, Experiment 1). Although these studies do not focus specifically on computer science and engineering, they do showcase that men are similarly turned off to STEM fields in general when they personally value communion and do not view these fields as affording opportunity for it.

### Consequences of having fewer communally-oriented men in STEM

An implication of the communal goal incongruity perspective is that it is not sufficient to focus exclusively on women: in order to change the stereotypes of STEM fields and the people that work within them, we need to understand why more communally-oriented men may not enter STEM fields and why those already in STEM may not express their communal values within these fields. More specifically, stereotypes about computer science and engineering as masculine and less communal can be challenged in several ways. First, one can incorporate the stories and expressed motivations and purposes of women and men in these fields in publicized narratives and views of STEM. Secondly, one can showcase the variability among men and women in the goals and reasons for why they do the work that they do.

Because the United States needs all of the trained and skilled STEM professionals possible to be innovative and competitive, it is important to consider communally-oriented men's decisions to enter STEM, pursue their communal goals in STEM, and leave STEM. These decisions might illuminate how stereotypes of STEM fields are perpetuated and opportunities to view STEM fields as communal are reduced. If communally-oriented men leave or never enter STEM fields, the remaining men in STEM may be less interested and driven by the communal nature of their work and, thus, continue to shape STEM environments in ways that do not afford communal goals for those who are motivated by them. Outsiders looking in might only see individuals driven primarily by agentic goals and infer that STEM does not afford communal goals and work. In this way, the stereotype is reified and perpetuated.

Past research has not directly tested these predictions, but existing evidence is suggestive. First, as highlighted in Cheryan et al.'s (2017) review, the point in the STEM pipeline with the greatest gender gap is at the early recruitment stage. Current data suggest that there are fewer women than men entering STEM college majors, but retention patterns of these male and female students are not markedly different (Miller and Wai, 2015). What can explain these recruitment differences? The communal goal incongruity perspective reviewed here suggests that prevalent noncommunal views of STEM, coupled with women's higher endorsement of communal goals, could result in the gender gaps found in recruitment to STEM disciplines. However, among men and women who value communal goals and have entered STEM, experiencing a noncommunal culture may result in similar rates of departure because they do not feel like they belong in the current climate of these fields, irrespective of their gender. Indeed, research finds that by fostering a more communal view of STEM fields, men overall are not demotivated to pursue these fields (Diekman et al., 2011; Clark et al., 2016), and communally-oriented men may become more motivated and invested in pursuing STEM.

A second related point is that just as cultural norms and stereotypes constrain women's career options, men's interests and decisions are similarly shaped by societal expectations and values. Croft et al.'s (2015) review finds that men are less likely to seek out communal roles because of gender stereotypes about who is and should be communal (e.g., Deaux and Major, 1987; Fiske et al., 2002; Wood and Eagly, 2002), concerns about how manly they will be perceived by others (e.g., Bosson and Vandello, 2011; Vandello and Bosson, 2012), and possible status loss, social sanctions, and discrimination they may face by fulfilling communal roles and goals (e.g., Heilman and Wallen, 2010; Moss-Racusin et al., 2010).

From these findings, it is possible that more communally-oriented men face substantial identity threat by openly discussing their communal goals and staying in STEM due to the discrepancies between personally and societally-valued goals for men. Multiple threats to one's self-image and self-worth exist in STEM fields; however, for communally-oriented men, they could experience the worry and uncertainty tied to social identity threat when they believe that they could be viewed and evaluated negatively due to their non-conformity to the perceived norms and values of STEM fields and their gender (e.g., Murphy and Taylor, 2012; Boucher and Murphy, 2017). Subtle or explicit cues that signal that their communal goals and interests could be limiting to their inclusion and success and are not expected of men can trigger social identity threat, and over time, these experiences can push communally-oriented men to leave for a more “identity safe” career option or shore up more of their agentic motivation and behavior that fits the current climate. One potential example of this progression would be communally-oriented male STEM majors gravitating away from academic science to professions like medicine where they can pursue their communal goals but still can align with societally-valued goals due to medicine's revered and agentic status. By understanding how men are steered away from communal roles or why they feel pressure to pursue more agentic goals, we can better understand how climates in STEM fields became and remain masculinized and illuminate points of intervention to improve the culture for both men and women.

Lastly, speaking specifically to how STEM stereotypes may be maintained or even strengthened by more communally-oriented men leaving STEM fields, we can draw links to classic social psychological research on stereotype change and maintenance. Without exposure to and presence of communally-oriented men in STEM, the distinction between women and men may be sharper, and the exemplars that come to mind for each may be more prototypical and extreme (e.g., Hamilton and Sherman, 1994). When individuals perceive these two groups are less varied, they are more willing to apply group stereotypes to individual members (e.g., Park and Rothbart, 1982; Park and Hastie, 1987; Park et al., 1991) and more likely to subtype individuals who do not fit the gender stereotypes of their group (e.g., Weber and Crocker, 1983; Rothbart and John, 1985). In this way, stereotypes about STEM fields remain unchanged. However, if STEM fields bring to mind men and women who vary in their reasons for pursuing STEM and their communal goals within STEM, stereotypes may change because it is harder to subtype counterstereotypic individuals. Highlighting how individuals across genders value communal goals at differing strengths, instead of perpetuating perceptions that a certain gender alone highly values communal goals, can lead to changes in the stereotypes of STEM fields and who fits within them.

This past work suggests important future research directions for demonstrating how noncommunal STEM stereotypes are perpetuated by the departure or absence of more communally-oriented individuals and how these stereotypes can be changed by efforts to recruit and retain these individuals. By exploring how communal goal incongruity affects men and women in potentially similar or different ways, we can advance our theoretical understanding of women's underrepresentation in STEM fields that have come close to gender parity and those that still have stark differences in women's participation like computing and engineering. Moreover, this research agenda can provide further insights into how lab-based evidence can be extended in the field and applied to efforts to make STEM fields more inclusive and welcoming to all groups.

## Part 3. Recommendations for reducing communal goal incongruity

The research literature on communal goal incongruity holds great promise for application and intervention. Balancing the nearly exclusive emphasis on agentic goals over communal goals can encourage and maintain interest and participation in STEM fields, particularly for those who value communion. Although highlighting the communal affordances in STEM has benefits, this perspective does not argue for an exclusive focus on these goals, because underrepresentation in STEM fields is multiply determined (e.g., Cheryan et al., 2017).

Creating and emphasizing the existing communal opportunities in fields like computing and engineering allows us to de-emphasize gender and racial categories while providing a way for majority and minority groups to come together around a shared motivational orientation. When the focus is on underlying motivational orientations, recruitment efforts can focus on shared values that transcend rather than reify categories. De-emphasizing group categories has additional benefits of reducing identity threat (e.g., Steele et al., 2002; Akcinar et al., 2011) and fostering a common ingroup identity that can result in bias reduction (e.g., Gaertner and Dovidio, 2000). As such, we conclude with recommendations for how to integrate this knowledge about communal goal affordances into practice.

A central theme of the following recommendations is that STEM fields *do* afford communal goals (see initiatives like Coders Without Borders and Engineers Without Borders), and people who value communal goals have a place within these fields. Our recommendations involve making these aspects of STEM more widely known and appreciated by (a) helping to change how STEM fields themselves are seen, (b) providing opportunities to act communally, and (c) widening the range of people seen working within these fields. Efforts toward these aims include creative ways of showcasing STEM, hands-on STEM experiences, and role models who share one's goals. Since past research has nicely delineated the efforts we currently know to be helpful for recruiting and retaining women in STEM (e.g., American Association of University Women, 2015; Cheryan et al., 2015), our suggestions stem from the consideration of our argument for how communal goal incongruity affects women and men and, thus, we focus on efforts that can disrupt noncommunal STEM stereotypes for all.

### Show students that STEM fields are communal

One way to increase interest in STEM is to change perceptions of what type of work these fields involve by making communal affordances known. Students do not often see the links to communal goals evident in STEM work (e.g., sustainability, food availability, medical innovations; Cunningham et al., 2005; National Academy of Engineering, 2008; Klotz et al., 2014). Therefore, complementing lessons of STEM concepts and skills with specific ways that these abilities and knowledge can improve the quality of lives or save lives can have great benefits for students (e.g., Freeman et al., 2014). Resources like Public Broadcasting Service's (PBS) STEM Education Resource Center include a wealth of accessible information about the real world applications of STEM work. After lessons that show STEM's communal possibilities, classroom activities that encourage students to make connections between STEM course material and their lives can further increase motivation and learning (e.g., Hulleman and Harackiewicz, 2009).

### Provide opportunities to act communally

In addition to conveying that STEM fields like computer science and engineering are communal, opportunities to practice collaboration and apply one's knowledge are needed. Participating in hands-on experiences that involve collaboration like solar car competitions and design challenges are one way to encourage students to act communally. Opportunities to work as a research assistant allows for more in-depth exposure to STEM work: these research opportunities show collaboration in action and familiarize students with the practical purposes of the work of which they are a part. As a successful example of implementing this in higher education, Harvey Mudd College provided research opportunities in computing after students' first year in college. This effort along with changes to curriculum and conference travel have resulted in the percentage of women graduating in computer science from 12% to around 40% in 5 years (Alvarado and Dodds, 2010; Alvarado and Judson, 2014).

### Provide role models who speak to how they are able to fulfill their communal goals through their work in STEM

To draw more female students into STEM, past work highlights the benefits of female role models (e.g., Dasgupta, 2011; Stout et al., 2011). Contact with older female students and female professors in STEM classes leads to more positive self-views and attitudes toward STEM and greater motivation to pursue STEM careers. Female role models are also particularly important to retaining women once they enter STEM; seeing the successes of other women in STEM can help prevent the threatening effects of stereotypes that shed doubt on women's competencies in these fields (Drury et al., 2011; Stout et al., 2011).

Alternatively, to recruit more communally-oriented individuals—both women and men—a role model's communal pursuits and traits may be more important than her or his gender. Both men and women who demonstrate how their STEM careers allow them to pursue communal goals can effectively convey that STEM provides valued opportunities to pursue communal goals (Clark et al., 2016). Furthermore, people may be interested in engaging with other-oriented STEM role models, even if they do not share a gender identity with the role model. Regardless of the advisor's gender, both men and women in STEM preferred to work with a hypothetical advisor who collaborated frequently over an advisor who spent much of his or her time working alone. However, these communally-oriented role models were perceived as particularly scarce in STEM relative to other fields, such as education or medicine (Fuesting and Diekman, 2017).

Thus, having people who work in STEM fields communicate the valued communal aspects of their work reduces communal goal incongruity and increases STEM interest and the likelihood of pursuing these fields (e.g., Klotz et al., 2014). For example, people may get interested in STEM because they want to improve the integrity of their community's roads and bridges, develop computer systems that help make aviation safer and more efficient, provide clean drinking water and better sanitation to improve health and safety, or even fight internet-based human trafficking. By encouraging people to share why they got into their STEM field and why they do the work they do, these individuals can validate the communal goals of students considering taking classes or majoring in STEM fields and can make these fields seem more communally oriented to current students and future generations of students. For much younger students, interviewing STEM professionals about what drew them to the field and what they find most fulfilling about their work can challenge stereotypes at even earlier ages.

In this way, it is important to shine more light on those who are pursuing their communal goals through their work in STEM. This can be accomplished by touring nearby labs and attending conferences that attract speakers from diverse backgrounds and with diverse motivations. Calling back to the changes Harvey Mudd College made to their computer science program, first year students were given the opportunity to attend the annual Grace Hopper Celebration of Women in Computing hosted by the Anita Borg Institute for Women and Technology. Events like this offer great opportunities to learn more about the diversity of people in certain STEM fields and what motivates their work. Attending conferences and visiting labs are helpful because they widen the view of who STEM professionals are and what they do, allow for networking with people at all levels of the field, and provide inspiring exemplars.

The existing evidence points to the benefits of varying students' views of who works in STEM and the goals that motivate them. Female and male mentors who demonstrate how they integrate their communal goals into their careers play a crucial role in recruiting and retaining communally-oriented women and men in STEM. This strategy might be especially helpful in fields like computer science and engineering that have the greatest female underrepresentation and strongest perceived incongruity with communal goals.

As this review details, there have been many advances in the understanding of patterns of underrepresentation for women in STEM fields like engineering and computer science. Motivational perspectives provide insight as to why people choose some fields over others, and considering underlying communal processes and noncommunal stereotypes brings together these multiple factors of climate and early experience that sustain gender gaps. By better integrating and publically acknowledging communal opportunities in computing and engineering, the stereotypic perceptions of these fields could gradually change, making computing and engineering more inclusive and welcoming to all.

## Author contributions

KB developed the review topic, conducted the literature review, led the writing of the paper, and revised the paper. MF helped develop the review topic, helped conduct the literature review, and contributed substantively to the writing and revising of the paper. AD helped develop the review topic and contributed substantively to the writing and revising of the paper. MM helped develop the review topic and contributed substantively to the writing and revision of the paper.

### Conflict of interest statement

The authors declare that the research was conducted in the absence of any commercial or financial relationships that could be construed as a potential conflict of interest.
